# Adult Lysophosphatidic Acid Receptor 1-Deficient Rats with Hyperoxia-Induced Neonatal Chronic Lung Disease Are Protected against Lipopolysaccharide-Induced Acute Lung Injury

**DOI:** 10.3389/fphys.2017.00155

**Published:** 2017-03-22

**Authors:** Xueyu Chen, Frans J. Walther, El H. Laghmani, Annemarie M. Hoogeboom, Anne C. B. Hogen-Esch, Ingrid van Ark, Gert Folkerts, Gerry T. M. Wagenaar

**Affiliations:** ^1^Laboratory of Neonatology, Division of Neonatology, Department of Pediatrics, Leiden University Medical CenterLeiden, Netherlands; ^2^Department of Pediatrics, Los Angeles Biomedical Research Institute at Harbor-UCLA Medical CenterTorrance, CA, USA; ^3^Department of Pharmacology, Utrecht Institute for Pharmaceutical Sciences, Utrecht UniversityUtrecht, Netherlands

**Keywords:** bronchopulmonary dysplasia, lung inflammation, adult rats, second hit response, aberrant lung development, LPA receptor, LPS

## Abstract

**Aim:** Survivors of neonatal chronic lung disease or bronchopulmonary dysplasia (BPD) suffer from compromised lung function and are at high risk for developing lung injury by multiple insults later in life. Because neonatal lysophosphatidic acid receptor-1 (LPAR1)-deficient rats are protected against hyperoxia-induced lung injury, we hypothesize that LPAR1-deficiency may protect adult survivors of BPD from a second hit response against lipopolysaccharides (LPS)-induced lung injury.

**Methods:** Directly after birth, Wistar control and LPAR1-deficient rat pups were exposed to hyperoxia (90%) for 8 days followed by recovery in room air. After 7 weeks, male rats received either LPS (2 mg kg^−1^) or 0.9% NaCl by intraperitoneal injection. Alveolar development and lung inflammation were investigated by morphometric analysis, IL-6 production, and mRNA expression of cytokines, chemokines, coagulation factors, and an indicator of oxidative stress.

**Results:** LPAR1-deficient and control rats developed hyperoxia-induced neonatal emphysema, which persisted into adulthood, as demonstrated by alveolar enlargement and decreased vessel density. LPAR1-deficiency protected against LPS-induced lung injury. Adult controls with BPD exhibited an exacerbated response toward LPS with an increased expression of pro-inflammatory mRNAs, whereas LPAR1-deficient rats with BPD were less sensitive to this “second hit” with a decreased pulmonary influx of macrophages and neutrophils, interleukin-6 (IL-6) production, and mRNA expression of *IL-6, monocyte chemoattractant protein-1, cytokine-induced neutrophil chemoattractant 1, plasminogen activator inhibitor-1*, and *tissue factor*.

**Conclusion:** LPAR1-deficient rats have increased hyperoxia-induced BPD survival rates and, despite the presence of neonatal emphysema, are less sensitive to an aggravated “second hit” than Wistar controls with BPD. Intervening in LPA-LPAR1-dependent signaling may not only have therapeutic potential for neonatal chronic lung disease, but may also protect adult survivors of BPD from sequelae later in life.

## Introduction

Very preterm infants are at high risk of developing severe respiratory distress syndrome (RDS) secondary to lung immaturity and surfactant-deficiency. Treatment with mechanical ventilation and supplemental oxygen interferes with postnatal lung development and damages the immature lung (Northway et al., 1967; Baraldi and Filippone, 2007; Philip, 2009; Bhandari, 2010; Mosca et al., 2011). Although, superb neonatal intensive care after preterm birth increases survival, it cannot prevent the development of neonatal chronic lung disease or bronchopulmonary dysplasia (BPD), which is the most common complication in children after preterm birth at <30 weeks of gestation (Jobe, 1999; Gien and Kinsella, 2011; Mosca et al., 2011; Jain and Bancalari, 2014). Severe BPD is characterized by arrested alveolar and vascular development, resulting in permanently enlarged alveoli, inflammation, and oxidative stress-induced lung damage and is complicated by vascular remodeling, which results in pulmonary arterial hypertension (PAH) and right ventricular hypertrophy (RVH; Northway et al., 1967; Mourani and Abman, 2015). Lung function in neonatal survivors of BPD is not only affected directly after birth, but also later in life, as these children are more susceptible to lower respiratory tract infections, wheezing and asthma, and PAH at relatively young ages (Bhandari and McGrath-Morrow, 2013; Carraro et al., 2013; Gough et al., 2014). Their quality of life and the burden to society of survivors of BPD is affected by frequent rehospitalization after discharge and BPD-associated pathology, such as poor neurodevelopmental outcome due to cerebral and cerebellar hemorrhages and hypoxic insults, delayed growth, and long-term gastro-intestinal problems (Dammann et al., 2004). Treatment of BPD is mainly symptomatic and focuses on gentler respiratory support to reduce neonatal lung damage and pharmacological interventions, such as diuretics for lung edema, caffeine to stimulate central respiratory drive, bronchodilators for life-threatening episodes of severe bronchospasm, and postnatal corticosteroids as anti-inflammatory agents (Baraldi and Filippone, 2007; Gien and Kinsella, 2011; Jain and Bancalari, 2014).

The Lysophosphatidic acid (LPA) receptor 1 (LPAR1) pathway has been considered a promising target for anti-fibrotic and anti-inflammatory therapy in adult and neonatal lung disease (Tager et al., 2008; Swaney et al., 2010; Rancoule et al., 2011; Chen et al., 2016). LPA receptors (LPAR1-6) are 7-transmembrane G protein-coupled receptors that are involved in many biological and pathological processes after binding to their ligand LPA (Yung et al., 2014). The glycerolipid LPA is generated enzymatically through hydrolysis of lysophosphatidylcholine by extracellular autotaxin (lysophospholipase D) or phospholipase A1 or A2. Increased LPA and autotaxin expression is associated with lung disease, including allergic asthma, fibrosis, and experimental BPD (Oikonomou et al., 2012; Park et al., 2013; Shim et al., 2015; Ackerman et al., 2016). Recently, Shim et al. (2015) demonstrated that neonatal exposure to hyperoxia in rat pups results in an increased expression and activity of the important LPA generating enzyme autotaxin and expression of the LPA receptors LPAR1 and LPAR3 compared to room air controls. These data strongly suggest that exposure of neonatal rat lungs to hyperoxia increases local LPA production and LPA-dependent signaling that may contribute to BPD pathology. The first identified LPA receptor with high affinity to LPA, LPAR1, or endothelial differentiation gene (EDG) family member 2 (EDG2), is expressed in many organs, including lung and heart (Choi et al., 2010). Reduced LPA-LPAR1-dependent signaling is associated with beneficial effects in many pulmonary diseases, including lung fibrosis, LPS-induced inflammation, bronchoconstriction, airway hyper-responsiveness, and (experimental) BPD (Toews et al., 2002; Tager et al., 2008; Swaney et al., 2010; Zhao et al., 2011; Chen et al., 2016), demonstrating the therapeutic potential of LPAR1 inhibition in adult and neonatal lung disease, including BPD.

To develop novel treatment strategies for BPD, animal models that mimic the clinical pathogenesis of BPD are mandatory (Buczynski et al., 2013; Hilgendorff and O'Reilly, 2015). Because rat and mouse pups are born at the saccular stage of lung development, similar to preterm infants at risk for developing BPD, alveolarization occurs after birth. Severe experimental BPD can be induced in newborn rat pups by exposure to 100% oxygen for 8 days, mimicking severe human BPD pathology including persistent alveolar enlargement, caused by arrested alveolar development and lung damage, inflammation, fibrosis and PAH (Wagenaar et al., 2004, 2013; de Visser et al., 2010; O'Reilly and Thébaud, 2014). In survivors of BPD, lung pathology may be complicated by an aggravated response toward a second hit. Mice with hyperoxia-induced BPD do not only exhibit an aggravated second hit response to viral infections, cigarette smoke, and bleomycin exposure, but their life span was also shorter (O'Reilly et al., 2008; McGrath-Morrow et al., 2011; Yee et al., 2011, 2013; Maduekwe et al., 2015). However, in rat models these aggravated responses toward a second hit remain to be elucidated. In a previous study, we demonstrated that blocking of LPAR1-dependent signaling, either by pharmacological intervention or by studying LPAR1-deficient rats, might be an effective treatment option for BPD by preventing inflammation-dependent lung injury. This was demonstrated by a reduced influx of macrophages and neutrophils, inflammatory cytokines production, and collagen deposition in the lung (Chen et al., 2016). Furthermore, the role of LPAR1-deficiency on the pathogenesis of a second hit response in survivors of BPD is unknown. This prompted us to investigate: [1] the role of LPAR1-deficiency on normal lung development, [2] the acute inflammatory response to LPS of adult rat survivors of BPD, and [3] the beneficial effects of LPAR1-deficiency on the development of a second hit response against LPS-induced acute lung injury in adult rats with hyperoxia-induced BPD.

## Materials and methods

### Animals

**Figure 1:**
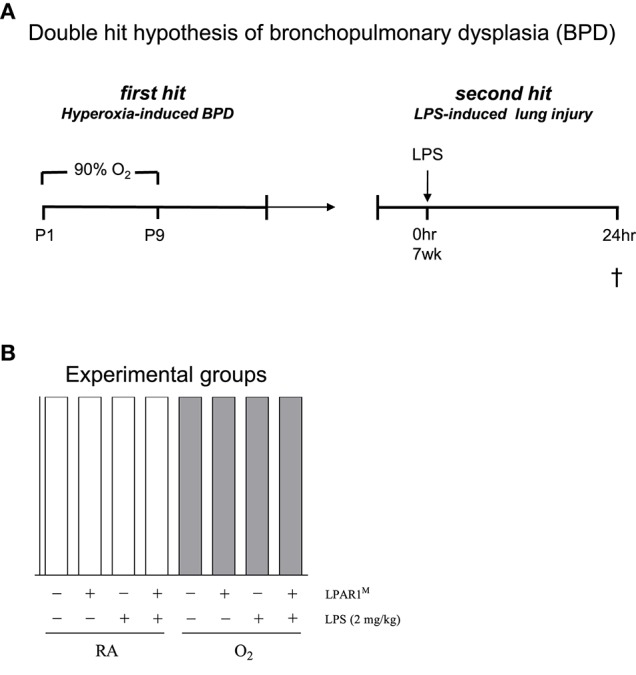
**The second hit hypothesis of bronchopulmonary dysplasia (BPD) was tested in Wistar control and Lysophosphatidic acid 1 (LPAR1) deficient rats: LPAR1^**M318R/M318R**^ (LPAR1^**M**^)**. Directly after birth, rat pups were exposed to 90% oxygen for 9 days (first hit) to induce experimental BPD **(A)**. At the age of 7 weeks, adult male rats were treated with LPS (2 mg kg^−1^) or 0.9% NaCl as a control by intraperitoneal injection to induce acute lung injury (second hit). In total, we studied 3 variables ([1] hyperoxia vs. room air, [2] LPAR1 deficiency vs. Wistar control, and [3] LPS vs. NaCl) in 8 experimental groups **(B)**. Rats with experimental hyperoxia-induced BPD are depicted by shaded bars and room air (RA) controls are depicted by white bars.

All animal experiments were approved by the Institutional Animal Care and Use Committee of the Leiden University Medical Center. LPAR1-mutant rats, generated by N-ethyl-N-nitrosourea mutagenesis, carry a missense mutation with a methionine instead of arginine at position 318 in the cytoplasmic 8th helix, resulting in a loss-of-function phenotype (van Boxtel et al., 2011; Chen et al., 2016). LPAR1-deficient (LPAR1^M318R/M318R^) rats were back-crossed for 11 generations on a Wistar rat background. For each experiment, newborn male Wistar or LPAR1-deficient (homozygous LPAR1^M318R/M318R^) rat pups from 3 to 5 litters were mixed together and equally assigned to 2 experimental groups: oxygen group (*N* = 12) and two room air (RA) groups (*N* = 6 each). First hit: the pups were exposed to 90% O_2_ from days 1 to 9 after birth (Figure 1A). All pups were fed by foster dams who were rotated daily to avoid oxygen toxicity: 24 h in 90% oxygen and 48 h in RA. Oxygen concentration, body weight, evidence of disease, and mortality were recorded daily. On day 9, the oxygen concentration was decreased to 75% and on day 10, pups were moved to room air. At the age of 7 weeks (body weight: 225–235 g), survivors of hyperoxia-induced BPD and RA controls were divided ad random into two groups: [1] second hit by intraperitoneal injection of 2 mg kg^−1^ of Lipopolysaccharides (LPS extracted from Pseudomonas aeruginosa, L9143, Sigma-Aldrich, St. Louis, MO, USA) in 1.5 ml 0.9% NaCl, or [2] injection with an equal volume of 0.9% NaCl (Figure 1A). Body weight of all rats from 8 experimental groups was measured 24 h after injection (Figures 1A,B). Hereafter, rats were anesthetized with isoflurane inhalation and sacrificed by exsanguination to collect lung tissue. From each animal, the right lung was frozen in liquid nitrogen for RT-PCR, and the left lung was fixed *in situ* by perfusion with 4% paraformaldehyde (PFA) for 6 min at a pressure of 11 cm H_2_O for histology (*N* = 7). In total, we studied 3 variables in 8 experimental groups (parameters (Figure 1B): [1] hyperoxia vs. room air, [2] LPAR1 deficiency vs. Wistar control, and [3] LPS vs. NaCl).

### Histology

Formalin-fixed, paraffin-embedded, 4 μm-thick lung sections were stained with hematoxylin and eosin, anti-ED-1 (monocytes and macrophages; diluted 1:5), anti-myeloperoxidase (MPO, RB-373-A1, Thermo Fisher Scientific, Fremont, CA, USA; diluted 1:1,500) and anti-von Willebrand factor (vWF, A0082, Dako Cytomation, Glostrup, Denmark; diluted 1:4,000). Primary antibody staining was visualized with Envision-HRP (K4001/K4003, Dako North America, Carpinteria, CA, USA) and NovaRed (SK-4800, Vector Laboratories, Burlingame CA, USA) as suggested by the supplier and counterstained briefly with hematoxylin. For morphometry of the lung, an eyepiece reticle with a coherent system of 21 lines and 42 points (Weibel type II ocular micrometer; Olympus, Zoeterwoude, The Netherlands) was used (Wagenaar et al., 2004). To investigate whether the enlarged alveoli in neonatal BPD persist into adulthood, we studied mean linear intercept (MLI), determined on hematoxylin and eosin stained lung sections, in 10 non-overlapping fields at a 200x magnification for each animal (Dunnill, 1962). The density of ED-1 positive monocytes and macrophages or MPO-positive neutrophilic granulocytes was determined in the alveolar compartment by counting the number of cells per field. Results were expressed as cells per mm^2^. Twenty fields in one section were studied at a 400x magnification. Capillary density was assessed in lung sections stained for vWF at a 200x magnification by counting the number of vessels per field. At least 10 representative fields per experimental animal were investigated. Results were expressed as relative number of vessels per mm^2^. Two independent researchers, who were blinded to the treatment strategy, performed lung morphometric analysis as described in previous studies (de Visser et al., 2010; Chen et al., 2015, 2016).

### Elisa

IL-6 levels were determined in lung tissue homogenates by ELISA (RRF600CKX, Antigenix America, Huntington Station, NY, USA) according to the instructions provided by the manufacturer.

#### Real-time RT-PCR

Real-time quantitative PCR was performed on a Light Cycler 480 (Roche, Almere, The Netherlands) at the Leiden Genome Technology Center (Leiden, The Netherlands), using first-strand cDNA synthesized from total RNA (SuperScript Choice System; Life Technologies, Breda, the Netherlands) and β-actin as a reference gene as previously described (Wagenaar et al., 2004). RNA was isolated from lung tissue homogenates (RNA-Bee, Tel-Test Inc, Bio-Connect BV, Huissen, the Netherlands), as described previously (Wagenaar et al., 2004, 2013). Primers are listed in Table 1.

**Table 1:** **Sequences of oligonucleotides for forward and reverse primers for real-time RT-PCR**.

**Gene product**	**Forward primer**	**Reverse primer**
IL-6	5′-ATATGTTCTCAGGGAGATCTTGGAA-3′	5′-TGCATCATCGCTGTTCATACAA-3′
CINC1	5′-GCACCCAAACCGAAGTCATA-3′	5′-GGGGACACCCTTTAGCATCT-3′
MCP-1	5′-ATGCAGTTAATGCCCCAGTCA-3′	5′-TTCTCCAGCCGACTCATTGG-3′
PAI-1	5′-AGCTGGGCATGACTGACATCT-3′	5′-GCTGCTCTTGGTCGGAAAGA-3′
TF	5′-CCCAGAAAGCATCACCAAGTG-3′	5′-TGCTCCACAATGATGAGTGTT-3′
HMOX1	5′-GACAGCATGTCCCAGGATTT-3′	5′-CTGGACACCTGACCCTTCTG-3′
β-actin	5′-TTCAACACCCCAGCCATGT-3′	5′-AGTGGTACGACCAGAGGCATACA-3′

#### Statistical analysis

Values are expressed as mean ± SEM. Differences between groups were analyzed by one-way ANOVA for independent samples, followed by Tukey's multiple comparison test, using the GraphPad Prism version 6 software package (San Diego, CA, USA). Differences at a *p* < 0.05 were considered statistically significant.

## Results

### Dosage finding for LPS-induced lung injury in adult rats

**Figure 2:**
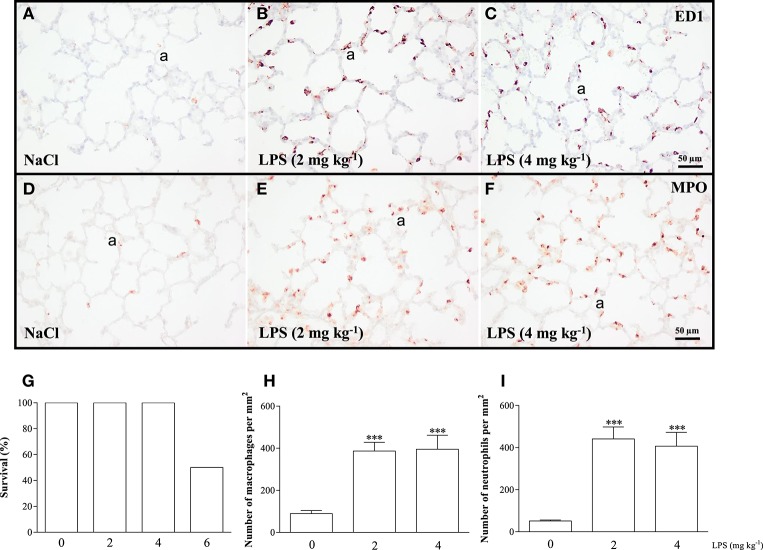
**The optimal dose of LPS to induce acute lung inflammation was investigated in a pilot experiment using adult Wistar rats, injected once with 0.9% NaCl or LPS at dosages of 2, 4, or 6 mg kg^**−1**^**. Twenty-four hours after injection, survival was recorded **(G)** and rats were sacrificed. Representative lung sections stained for the macrophage marker ED1 **(A–C)** and neutrophilic granulocyte marker MPO **(D–F)** were quantified in **(H)** (macrophages) and **(I)** (neutrophils) in rats injected with 0 (NaCl control), 2 or 4 mg kg^−1^ of LPS. Values were expressed as mean ± SEM, *N* = 6. ^***^p < 0.001 vs. NaCl control. a, alveolus.

We performed a pilot study to determine the optimal dose of LPS to induce acute lung inflammation. Male Wistar rats (mean 230 g) were injected intraperitoneally with 2, 4, or 6 mg kg^−1^ of LPS or 0.9% NaCl. Survival and influx of macrophages and neutrophils into the lung were quantified as read-outs (Figure 2). Because 6 mg kg^−1^ of LPS led to a mortality rate of 50%, this dosage of LPS was considered inappropriate (Figure 2G). All rats injected with 0.9% NaCl, or 2 or 4 mg kg^−1^ LPS survived. Because the inflammatory response, determined by the massive LPS-induced influx of ED1-positive macrophages (Figures 2B,C,H) and MPO-positive neutrophils (Figures 2E,F,I) was similar after administration of LPS doses of 2 and 4 mg kg^−1^, we decided that 2 mg kg^−1^ of LPS was the optimal dose.

### LPAR1-deficiency protects against neonatal hyperoxia-induced mortality

**Figure 3:**
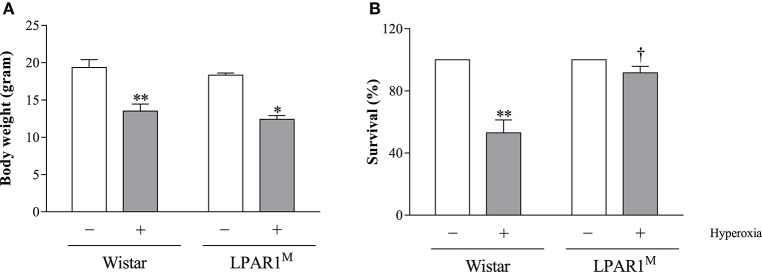
**Body weight (A)** and survival **(B)** of Wistar and LPAR1-deficient (LPAR1^M318R/M318R^) pups after 8 days in RA (open bars) and exposure to hyperoxia (shaded bars). Values were expressed as mean ± SEM, *N* = 7–10. ^*^p < 0.05 and ^**^p < 0.01 vs. their own RA control. ^*†*^*p* < 0.05 vs. Wistar controls with neonatal chronic lung disease or BPD. Three independent experiments were performed.

Exposure to hyperoxia for 8 days significantly decreased body weight of Wistar pups (mean 13.5 g; *p* < 0.01) and LPAR1 deficient pups (12.4 g; *p* < 0.05), compared to their room air-exposed controls (Wistar: 19.3 g and LPAR1: 18.3 g; Figure 3A). All pups survived in room air. Mortality in Wistar pups exposed to hyperoxia for 8 days was twice as high as in RA controls (*p* < 0.01) and also exceeded mortality in LPAR1-deficient pups (*p* < 0.05 compared to Wistar pups exposed to hyperoxia; Figure 3B).

### LPAR1-deficiency results in emphysema in adult rats

**Figure 4:**
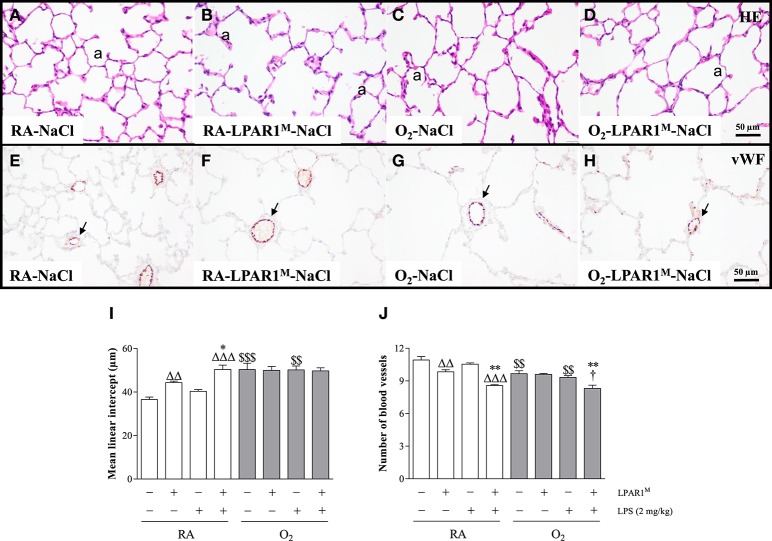
**Representative HE-stained (A–D)** and von Willebrand factor (vWF)-stained **(E–H)** lung paraffin sections of adult Wistar rats **(A**,**C**,**E**,**G)** and LPAR1-deficient (LPAR1^M318R/M318R^) rats **(B**,**D**,**F**,**H)** injected with 0.9% NaCl **(A–D)** or 2 mg kg^−1^ of LPS **(E–H)**. Quantifications of mean linear intercept **(I)** and number of pulmonary vessels **(J)** were determined on sections in Wistar and LPAR1-deficient rats with neonatal chronic lung disease or BPD (shaded bars) or RA controls (white bars). Values are expressed as mean ± SEM, *N* = 7. ^*^p < 0.05 and ^**^p < 0.01 vs. their own NaCl controls. ^ΔΔ^*p* < 0.01 and ^ΔΔΔ^*p* < 0.001 vs. RA-NaCl control, *p* < 0.05 vs. Wistar rats with BPD. ^$$^*p* < 0.01 and ^$$$^*p* < 0.001 vs. their own RA controls. Three independent experiments were performed. a, alveolus. Arrows in panels **(E–H)** indicate vWF-stained blood vessels.

Seven weeks old adult LPAR1-deficient rats had enlarged alveoli (Figure 4B) as shown by an increased mean linear intercept (MLI; 1.2-fold; *p* < 0.01; Figure 4I) and decreased vascularization (1.1-fold; *p* < 0.01; Figures 4F,J) compared to Wistar controls (Figures 4A,E,I,J). LPS had no effect on alveolar enlargement and vascularization in Wistar controls, but aggravated the increase in alveolar size (*p* < 0.05) and reduction in vascularization (*p* < 0.01) in LPAR1-deficient rats compared to RA-LPAR1^M^-NaCl controls.

### Hyperoxia-induced neonatal emphysema and reduced angiogenesis persists into adulthood

Adult survivors of BPD showed alveolar enlargement and reduced vascularization, demonstrated by an increased MLI (1.4-fold, *p* < 0.001; Figures 4C,I) and a decreased number of blood vessels (1.1-fold, *p* < 0.01; Figures 4G,J), respectively compared to RA-exposed controls (Figures 4A,E), demonstrating persistent emphysema and reduced vascularization in survivors of BPD. LPS administration had no impact on MLI (Figure 4I), but reduced pulmonary vessel density by 1.1-fold (Figure 4J), compared to LPS-stimulated Wistar (*p* < 0.05) or NaCl-treated LPAR1-deficient rats with hyperoxia-induced BPD (*p* < 0.01; Figures 4D,H).

### LPAR1-deficiency protects against LPS-induced pulmonary influx of inflammatory cells and IL-6 expression

**Figure 5:**
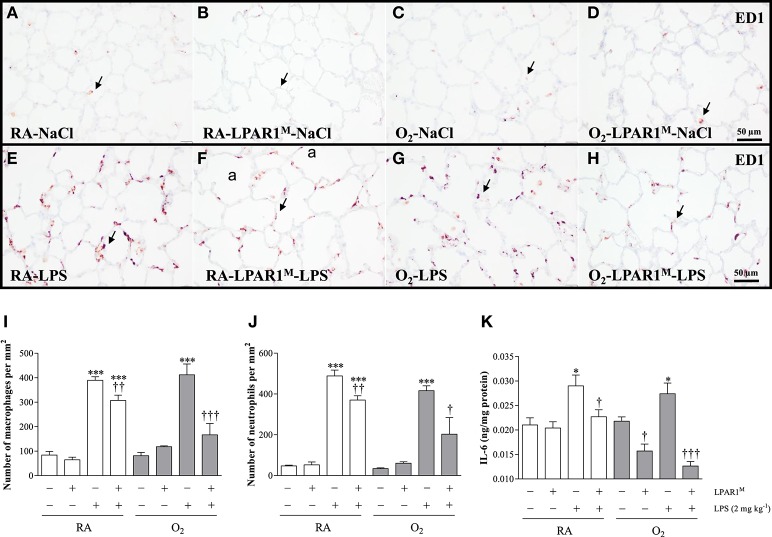
**Representative lung paraffin sections stained for the macrophage marker ED1 in rats injected with 0.9% NaCl (A–D)** or 2 mg kg^−1^ of LPS **(E–H)**. Quantification of macrophages **(I)** and neutrophils **(J)** on sections and IL-6 ELISA **(K)** in lung homogenates were determined in Wistar and LPAR1-deficient (LPAR1^M318R/M318R^) rats with neonatal chronic lung disease or BPD (shaded bars) or RA controls (white bars). Values are expressed as mean ± SEM, *N* = 7. ^*^p < 0.05 and ^***^p < 0.001 vs. their own NaCl controls. ^*†*^*p* < 0.05, ^*††*^*p* < 0.01, and ^*†††*^*p* < 0.001 vs. Wistar rats with BPD. Three independent experiments were performed. a, alveolus. Arrows indicate ED1-positive macrophages.

Adult Wistar control and LPAR1-deficient rats were raised in RA after birth and developed a significant inflammatory response toward LPS. This was demonstrated by an influx of macrophages (*p* < 0.001; Figures 5E,F,I), neutrophils (*p* < 0.001; Figure 5J), and increased expression of interleukin 6 at the protein level IL-6; *p* < 0.05; Figure 5K), compared to NaCl-treated controls (Figures 5A,B). The LPS-induced pulmonary influx of macrophages (1.3-fold; *p* < 0.01) and neutrophils (1.6-fold; *p* < 0.01) and IL-6 protein expression (1.3-fold; *p* < 0.05) was significantly reduced in LPAR1-deficient rats compared to LPS-stimulated Wistar control rats.

### LPAR1-deficiency attenuates the adult LPS-induced inflammatory second hit response

Neonatal exposure to hyperoxia led to a transient inflammatory response characterized by an influx of macrophages and neutrophils in neonatal rat lung, which did not persist into adulthood (Figures 5C,D,I,J). Administration of LPS did not aggravate the influx of macrophages (Figures 5G,I) and neutrophils (Figure 5J), and IL-6 expression (Figure 5K) in survivors with BPD compared to RA-exposed Wistar controls. However, in adult LPAR1-deficient rats with BPD the LPS-induced influx of macrophages (Figures 5H,I) and neutrophils (Figure 5J) in lung tissue sections and the level of IL-6 in lung tissue homogenates (Figure 5K) were lower compared to Wistar rats with BPD: 2.4-fold, *p* < 0.001 for macrophages; 2.1-fold, *p* < 0.05 for neutrophils; and 2.2-fold, *p* < 0.001 for IL-6 expression. In addition, the basal level of IL-6 in the BPD lung was higher in Wistar rats than in LPAR1-deficient rats (1.4-fold, *p* < 0.05; Figure 5K).

### LPAR1-deficiency attenuates the aggravated pro-inflammatory and pro-coagulant second hit response toward LPS in adult rats with BPD at the transcriptional level

**Figure 6:**
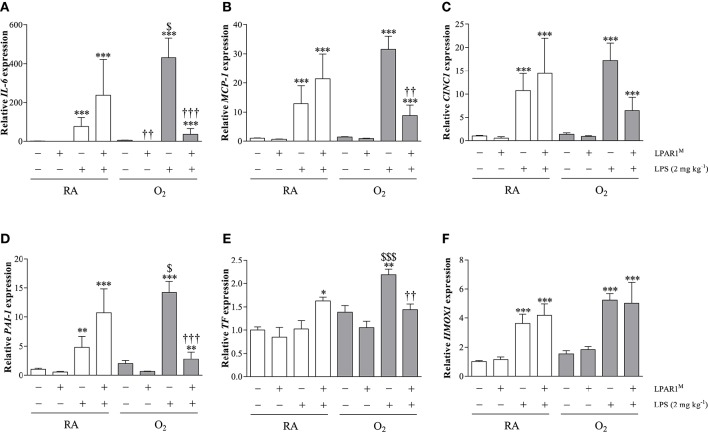
**Relative mRNA expression of interleukin 6 (IL-6; A)**, monocyte chemoattractant protein-1 (MCP-1; **B**), cytokine-induced neutrophil chemoattractant 1 (CINC1; **C**), plasminogen activator inhibitor-1 (PAI-1; **D**), tissue factor (TF; **E**) and Heme Oxygenase 1 (HMOX1; **F**) in lung homogenates of adult Wistar and LPAR1-deficient (LPAR1^M318R/M318R^) rats with neonatal chronic lung disease or BPD (shaded bars) or Wistar RA controls (white bars), injected with 0.9% NaCl or 2 mg kg^−1^ of LPS, using β-actin as a house keeping gene reference. Values are expressed as mean ± SEM, *N* = 7. ^*^p < 0.05, ^**^p < 0.01, and ^***^p < 0.001 vs. their own NaCl controls. ^*††*^*p* < 0.01 and ^*†††*^*p* < 0.001 vs. Wistar rats with BPD. ^$^*p* < 0.05 and ^$$$^*p* < 0.001 vs. Wistar RA control rats. Three independent experiments were performed.

The basal mRNA expression (Figure 6) of *IL-6* (Figure 6A), *MCP-1* (Figure 6B), *CINC1* (Figure 6C), *PAI-1* (Figure 6D), *tissue factor* (*TF*; Figure 6E), and *Heme Oxygenase 1* (*HMOX1*; Figure 6F) was comparable in Wistar rats with and without BPD and in LPAR1-deficient rats with BPD except for *IL-6*. Basal *IL-6* expression was lower in LPAR1-deficient rats with BPD (*p* < 0.01), compared to adult Wistar controls with BPD. In control Wistar rats, LPS stimulated mRNA expression of *IL-6* (75.9-fold; *p* < 0.001), *MCP-1* (12.9-fold; *p* < 0.001), *CINC1* (10.7-fold; *p* < 0.001), *PAI-1* (4.8-fold; *p* < 0.001), and *HMOX1* (3.6-fold; *p* < 0.001) compared to NaCl-treated Wistar controls. LPS-induced mRNA expression of *IL-6, MCP-1, CINC1*, and *PAI-1* in adult LPAR1-deficient and in Wistar control rats was not significantly different, but there was a tendency toward higher levels in LPAR1-deficient rats.

Neonatal exposure to hyperoxia aggravated the LPS-induced second hit response in adult rat lung for *IL-6* (5.7-fold; *p* < 0.05), *PAI-1* (3.0-fold; *p* < 0.05), and *TF* (2.1-fold; *p* < 0.001), whereas *MCP-1 and CINC1* showed a tendency toward higher levels, compared to LPS-stimulated RA Wistar controls. The second hit response toward LPS was prevented in LPAR1-deficient rats with BPD for *IL-6* (12.0-fold; *p* < 0.001), *MCP-1* (3.6-fold; *p* < 0.01), *PAI-1* (5.1-fold; *p* < 0.001), and *TF* (1.5-fold; *p* < 0.01), whereas *CINC1* showed a tendency toward lower levels than LPS-stimulated Wistar rats with BPD. The LPS response toward *HMOX1* expression was not affected by exposure to hyperoxia or LPAR1-deficiency.

## Discussion

This study demonstrates that [1] acute lung injury in adulthood induces an aggravated second hit response in rats with neonatal hyperoxia-induced BPD, [2] LPAR1-deficiency results in emphysema and reduced vascularization in adult rats, and [3] LPAR1-deficient rats with BPD are protected against the exacerbated second hit response induced by LPS. The persistence of emphysema and reduced vascularization resulting from neonatal exposure to hyperoxia is consistent with our previous finding in the same BPD rat model (de Visser et al., 2012).

We found that LPAR1-deficiency leads to alveolar enlargement or emphysema and decreased pulmonary vessel density in adult rats. This finding confirms observations in LPAR1 knockout mice showing that aberrant alveolar development caused by reduced secondary septation during early lung development was visible from 3 weeks after birth until adulthood (Funke et al., 2016). This explains why we did not observe alveolar enlargement in our previous study, in which the effects of LPAR1-deficiency on aberrant lung development were limited to the early neonatal period (Chen et al., 2016). LPAR1-deficiency may contribute to arrested alveolar development and reduced secondary septation by remodeling of elastic fibers, resulting in a reduction of disorganized septal fibers (Funke et al., 2016). The adverse effect of LPAR1 deficiency on alveolar development may limit the clinical usage of LPAR1 inhibitors for BPD. However, in LPAR1 deficient rats LPA-LPAR1 dependent signaling is reduced during pre- and postnatal development, whereas inhibition of the LPA-LPAR1 pathway is likely only needed during the relatively short pulmonary injurious period of mechanical ventilation with supplemental oxygen. Therefore, the adverse effect of a potent LPAR1 blocker on adverse alveolar development will probably be less severe when used postnatally in premature infants with BPD, thereby limiting the adverse effects on emphysema development in adult survivors of BPD.

Exposure to hyperoxia or air pollution induces oxidative stress and generates cytotoxic reactive oxygen species (ROS) in lung cells. ROS, either generated directly or indirectly by activated inflammatory cells, can cause epithelial and endothelial cell injury and death by activation of complex signal transduction pathways and epigenetic factors (Hagood, 2014; Ni et al., 2015; Porzionato et al., 2015). Oxidative stress after LPS stimulation was demonstrated in this study by an increase in expression of *HMOX1*. In neonatal chronic lung disease (BPD), adult chronic obstructive pulmonary disease (COPD), and LPS-induced acute lung injury oxidative stress activates multiple signal transduction pathways that belong to the mitogen-activated protein kinases (MAPK) family, including extracellular signal-regulated kinase (ERK1/2), C-Jun-terminal protein kinase (JNK1/2) and p38 kinase, and nuclear factor kappa-light-chain-enhancer of activated B cells (NF-κB) (Ryter et al., 2007; Zuo et al., 2012; Porzionato et al., 2015). Activation of ERK protects lung cells against hyperoxia-induced injury and death through stimulation of DNA repair and anti-oxidant mechanisms, whereas activation of JNK1/2, p38 kinase and NF-κB is associated with adverse effects, including tissue damage via necrosis and apoptosis, TGF-β-induced aberrant alveolarization and activator protein-1-induced inflammation. In addition, ROS may contribute to disease pathology by epigenetic effects via histone modification, RNA interference, and DNA methylation (Hagood, 2014). In mammals, neonatal oxidative stress, induced by prolonged exposure to hyperoxia, disrupts alveolar and vascular development of the immature lung, which results in enlarged alveoli, increased septal thickness, pulmonary influx of inflammatory cells, including macrophages and neutrophils, and vascular remodeling (Madurga et al., 2013).

In agreement with our previous studies, we found that neonatal hyperoxia-induced emphysema persists into adulthood (de Visser et al., 2012) and confirm similar observations in mice with BPD (O'Reilly et al., 2008; McGrath-Morrow et al., 2011). Furthermore, this study shows for the first time in rats that the inflammatory second hit response is aggravated in survivors of BPD after administration of LPS. These data confirm previous observations in mice with BPD that showed an exacerbated second hit response to exposure to cigarette smoke-induced emphysema, viral infection, and bleomycin-induced lung fibrosis (O'Reilly et al., 2008; McGrath-Morrow et al., 2011; Yee et al., 2013). Collectively, these findings demonstrate lung hyper-responsiveness to viral and toxic insults in rodents with hyperoxia-induced BPD.

The second hit response in adult rats with BPD was clearly demonstrated at the mRNA level by an increased expression of the pro-inflammatory marker *IL-6*, the procoagulant gene *TF* and the regulator of fibrinolysis *PAI-1*, but was less pronounced or even absent at the protein level. This discrepancy in second hit response at the protein level between our study and the previously published mouse studies may be attributed to differences in species, injurious trigger, sampling, and a more pronounced effect at the transcriptional level due to the experimental setup. We studied acute lung pathology at 24 h after LPS stimulation with a relatively high dose of LPS (2 mg kg^−1^) to induce lung injury, whereas in mice chronic lung diseases were studied for weeks, including cigarette smoke-induced emphysema, viral infection, and bleomycin-induced lung fibrosis (O'Reilly et al., 2008; McGrath-Morrow et al., 2011; Yee et al., 2013).

The LPA-LPAR1 pathway has been regarded as a promising target for anti-fibrotic and anti-inflammatory therapy (Tager et al., 2008; Swaney et al., 2010; Rancoule et al., 2011). We demonstrated that LPAR1-deficiency reduces the influx of inflammatory cells and IL-6 after a LPS challenge in adult rats and, more effectively, the second hit response against LPS in adult rats with neonatal chronic lung disease or BPD, in which not only the influx of inflammatory cells and IL-6 was strongly reduced, but also the mRNA expression of *IL-6, MCP-1, CINC1, TF*, and *PAI-1*. In previous work, we found that pharmacological blocking of the LPA-LPAR1 signaling pathway with Ki16425 protected against BPD in neonatal rat pups (Chen et al., 2016). However, the role of LPA/LPAR1-dependent signaling in pulmonary inflammation is still controversial. Although, LPS increases LPA in plasma (Zhao et al., 2011; Awada et al., 2014; Mouratis et al., 2015), suggesting a role for LPA-LPAR1 signaling in the LPS response, *in vitro* and *in vivo* data demonstrate that both stimulation (Murch et al., 2007; Fan et al., 2008; He et al., 2009; Zhao et al., 2012) and inhibition (Zhao et al., 2011; Chen et al., 2016) of this pathway result in a reduced inflammatory response. Here, we found that the beneficial effects of LPAR1-deficiency are not only limited to the neonatal period (Chen et al., 2016), because LPAR1-deficiency ameliorated the LPS-induced second hit response in adult survivors of BPD. LPAR1-deficient rats showed a reduced infiltration of macrophages and neutrophils into the lung, IL-6 protein expression and *IL-6, MCP-1, CINC1, PAI-1*, and *TF* mRNA expression in the lung during a second hit. The disparities in the effect of LPA-LPAR signaling on inflammation may be due to differences in gene regulation at the transcriptional and post-transcriptional level, experimental setup, including differences in inflammatory parameters studied at different time points, LPA receptors, cell types, and background (Chu et al., 2015).

LPAR1 is a 7-transmembrane G protein-coupled receptor (GPCR) that couples with three types of G proteins after binding to its ligand LPA: Gα12/13, Gαq/11, Gαi/o, resulting in the initiation of many downstream signaling cascades, including the Rho-ROCK, phospholipase C, Ras, P38-MAPK, Akt, and NF-κ B pathway and inhibition of adenylyl cyclase (AC) (Lin et al., 2010; Zhao et al., 2011; Yung et al., 2014). Many of these pathways are also affected in the pathogenesis of hyperoxia-induced neonatal lung injury, including AC-, activator protein 1 (AP1)-, P38 MAPK-, NF-κB-, and akt-dependent signaling (Perkowski et al., 2003; Wagenaar et al., 2004; Porzionato et al., 2015). This suggests that the beneficial effects of LPAR1 inhibition on ROS- and inflammation-driven lung injury and fibrosis is very complex and may be explained by reduced LPAR1-dependent signaling via multiple signal transduction pathways or cAMP via activation of AC. The beneficial effect of LPAR1-deficiency on the LPS response in rats with BPD is further complicated by the biological response of LPAR1-deficiency on two different injurious stimuli, inflicted at two different time-points, i.e., hyperoxia-induced lung injury in the neonatal period and LPS-exposure during adulthood. Several studies have provided preliminary data on the mechanism by which LPA-LPAR blocking protects against lung injury after a single injurious response caused by either hyperoxia or LPS. Hyperoxia-induced lung inflammation is associated with an increased expression of autotoxin, LPAR1, and LPAR3 in neonatal rats (Shim et al., 2015) and is stimulated by LPA-dependent activation of natural killer T (NKT) cells in adult mice (Nowak-Machen et al., 2015). Furthermore, blocking of LPA/LPAR-dependent signaling reduces LPS-induced lung injury by attenuating the activation of p38 MAPK and NF-κB and IL-6 production in adult mice (Zhao et al., 2011). However, mechanistic data on the response of a second hit by LPS after hyperoxic lung injury are lacking. Therefore, additional studies are needed to elucidate the complex mechanisms by which LPAR1 blocking protects against LPS-induced lung injury in rats with BPD.

In summary, the current study shows that, similar to humans and mice, lungs of adult rats that survived BPD are hyper-responsive to a second hit. Furthermore, deficiency of LPAR1 attenuates the inflammatory response to a second hit in adult rats with BPD. These findings extend our previous work by showing that LPAR1 blocking not only protects against neonatal hyperoxia-induced BPD, but also has additional long-term beneficial effects on the response to insults later in life. Therefore, blocking the LPA-LPAR1 signaling pathway may be a promising pharmaceutical target for the treatment of BPD and prevention of its sequelae later in life.

## Author contributions

FW, GF, and GW participated in research design. XC, EL, AH, AH-E, and IvA conducted experiments. XC, EL, AH, IvA, and GW performed data analysis. XC, FW, GF, and GW wrote or contributed to the manuscript.

## Funding

The authors gratefully acknowledge financial support by the National Institutes of Health through grants R01 HL092158 and R01 ES015330 (FW), by the China Scholarship Council through grant no. 201407720025 (XC), and by Chiesi Pharmaceuticals BV (GW and FW).

### Conflict of interest statement

The authors declare that the research was conducted in the absence of any commercial or financial relationships that could be construed as a potential conflict of interest.
